# Supplementary figures and images for: A reproducible approach to high-throughput biological data acquisition and integration
Source: PeerJ. 2015 Mar 31;3:e791. doi: 10.7717/peerj.791 (PMC4493686; doi:10.7717/peerj.791)

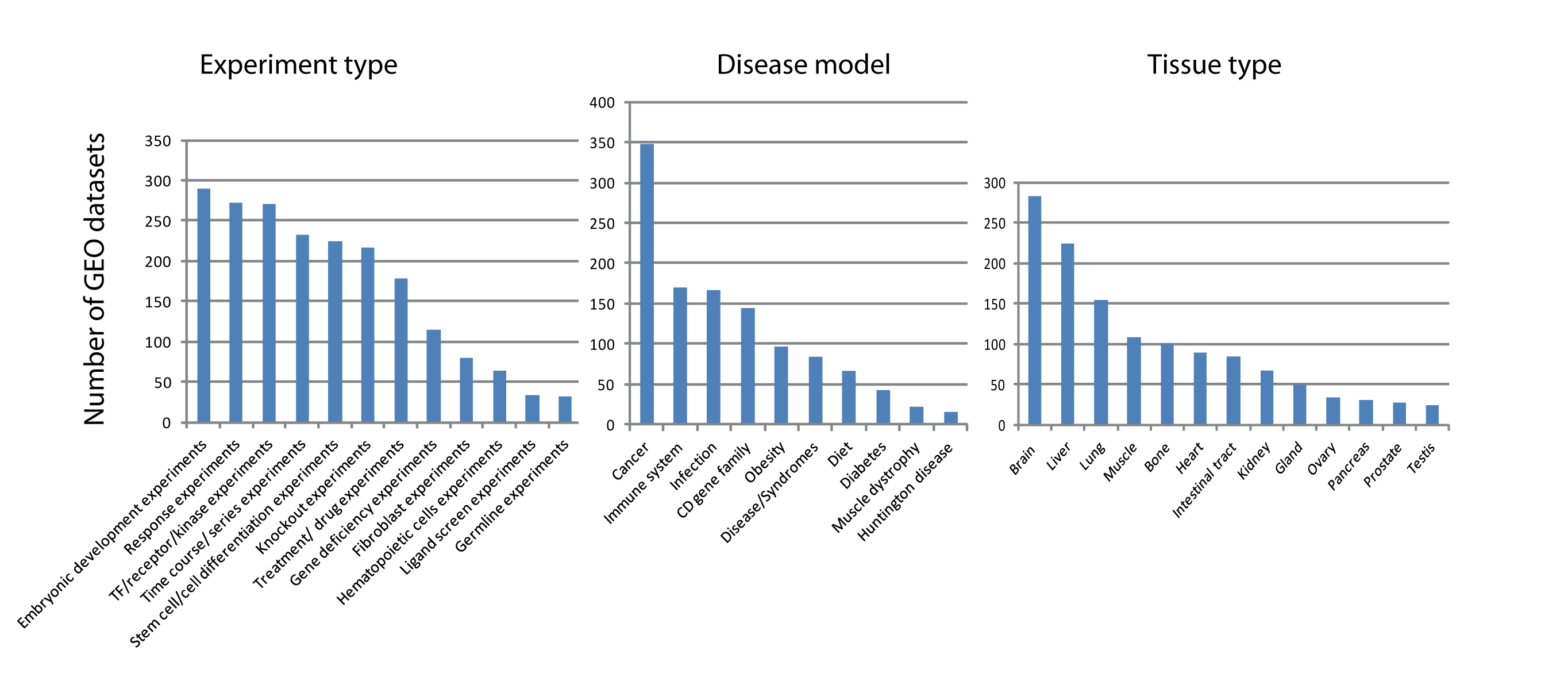

Supplement: Figure S1 — We screened ARepA’s murine metadata by matching different experimental types, disease models, or tissue types and obtained different lists of matching datasets for which the number of datasets are visualized in this figure. [file peerj-03-791-s001.png]
