# Supplementary material for: A reproducible approach to high-throughput biological data acquisition and integration
Source: PeerJ. 2015 Mar 31;3:e791. doi: 10.7717/peerj.791 (PMC4493686; doi:10.7717/peerj.791)
Supplement: Text S1 [file peerj-03-791-s007.docx]

**Supplementary Text 1: Details of ARepA’s case studies**

**Case Study 1: Human Prostate cancer**

- 1. **Retrieve data**
- Get all metadata (pkl files) downloaded for human (Homo sapiens) *a priori*.

Metadata query that searches for all datasets that are prostate cancer specific using the unpickle.py script in the source directory:

$ for i in *.pkl; do python /arepa/src/unpickle.py < $i| grep “prostate cancer”; done

This query resulted in 10 human prostate cancer specific gene expression datasets as listed in Supplementary Table S1 as of 02/2013.

- Get these datasets from ARepA with activated Sleipnir option (default: “off” -> change into “on”) in order to obtain co-expression networks as “dat/dab” files.
  1. **Meta-analysis**

The script *casestudy1.py* can be executed in order to reproduce the case study results:

$ python casestudy1.py <path to pcl files (step 1)> casestudy1_querygenes.txt

- Meta-analyze these single co-expression networks into a single prostate cancer specific functional network using unsupervised data integration averaging across normalized co-expression values (z-scores) using *Combiner* in Sleipnir with default setting.

$ Combiner -t dat -o <combined.dab> -n <data.dab>*

Documentation: <http://libsleipnir.bitbucket.org/Combiner.html>

Reference: Huttenhower et al. Genome Res. 2009 Jun;19(6):1093-106

- Extract a high-confidence subgraph based on the probabilistic graph search algorithm. This starts from a user-defined set of query genes and identifies *k* additional neighbours in the network that are connected with high confidence to the original query genes. Here, we defined a query gene set of twelve genes from the NFκB signaling pathway in BioCarta (Supplementary Table 4) and a neighbourhood size of *k*=10 using *Dat2Graph* in Sleipnir with default setting.

$ Dat2Graph -i <data.dab> -t dat -q <genes.txt> -k 10

Documentation: <http://libsleipnir.bitbucket.org/Dat2Graph.html>

Reference: Huttenhower et al. Genome Res. 2009 Jun;19(6):1093-106, Myers et al. Genome Biology 2005, **6**:R114

- Result: High-confidence NFκB signalling network containing 22 genes in total as illustrated in Figure 2.

**Case Study 2: Mouse meta-analysis**

1. **Retrieve data**

- Get all metadata (pkl files) downloaded for mouse (Mus musculus) *a priori*.
- Metadata query that are germ-free and tissue specific using the unpickle.py script in the source directory:

$ for i in *.pkl; do python /arepa/src/unpickle.py < $i| grep “germ-free” | grep “wild type” | grep “intestinal tract”; done

This query resulted in 6 case-control datasets from germ-free versus wild type mouse as listed in Supplementary Table 2 as of 02/2013.

- Get these 6 datasets from ARepA with default setting to obtain normalized and standardized expression datasets as “pcl” files.

1. **Meta-analysis**

The scripts *casestudy2_computeDE.py, casestudy2_computeMean.py,* and *casestudy2_forestplpots.R* can be executed in order to reproduce the case study results.

- Compute log fold changes and confidence intervals (2.5% and 97.5%) for all genes between germ-free gut and wild type gut within each dataset using the R/limma package, resulting in ~3,600 differentially expressed genes (*casestudy2_computeDE.py, casestudy2_computeMean.py)*.

Reference: Smith et al. Springer 2005: 397-420

- Perform a meta-analysis using the R/metafor package on the six datasets by applying a random-effects model on the fold changes with default options, fitting the model with the restricted maximum-likelihood estimator (REML) (*casestudy2_forestplpots.R)*. The false discovery rate was controlled by the Benjamini-Hochberg method.

Reference: Viechtbauer et al. 2010 Journal of Statistical Software 36(3): 1-48.

- Test all genes and their resulting meta-p-values for gene set enrichment (GSEA) in KEGG and BioCarta pathways.
- Result: 2 pathways that were significantly enriched for upregulated genes under germ-free conditions (using 1,000 permutations), while 15 pathways were enriched for downregulated genes as shown in Supplementary Table 3.

**Case Study 3: Bacterial data integration**

1. **Retrieve data**

- Get all gene expression data (GEO) and gene interaction data (IntAct, MPIDB, RegulonDB, Bacteriome and STRING, Table 2A) as of 02/2013 from ARepA with activated Sleipnir option (default: “off” -> change into “on”) in order to obtain co-expression networks as “dat/dab” files for *Bacillus subtilis, Escherichia coli* and *Pseudomonas aeruginosa*. Use UniRef90 as the output gene identifier for all bacteria.
  - - - GEO as of 02/2013, refer to Supplementary Table S2
      - Intact version 2013-01-30
      - MPIDB **Release 2009-11-18**
      - RegulonDB Release 8.1
      - Bacteriome as of 02/2013
      - STRING v9.0

1. **Meta-analysis**

The script *casestudy3.py* can be executed in order to reproduce the case study results; for each bacterium the script is executed individually, using the appropriate downloaded data files (pcl) and query genes (*genes_secretion_ecoli_KOs.txt, genes_secretion_bacillus_KOs.txt, genes_secretion_pseudomonas_KOs.txt*):

$ python casestudy3.py <path to pcl files (step 1)> genes_secretion_ecoli_KOs.txt

- Within each bacterium, integrate these data into a species-specific functional network using an unsupervised data integration averaging across normalized co-expression values (z-scores) using *Combiner* in Sleipnir with default setting.

$ Combiner -t dat -o <combined.dab> -n <data.dab>*

Documentation: <http://libsleipnir.bitbucket.org/Combiner.html>

Reference: Huttenhower et al. Genome Res. 2009 Jun;19(6):1093-106

- Extract a high-confidence subgraph based on the probabilistic graph search algorithm. This query started from a user-defined set of genes of the sec and tat genes and genes from the Type I, Type II, Type III, Type V, and Type VI secretion systems (Supplementary Table 4B) and used a neighbourhood size of *k*=5 using *Dat2Graph* in Sleipnir with default setting.

$ Dat2Graph -i <data.dab> -t dat -q <genes.txt> -k 5

Documentation: <http://libsleipnir.bitbucket.org/Dat2Graph.html>

Reference: Huttenhower et al. Genome Res. 2009 Jun;19(6):1093-106, Myers et al. Genome Biology 2005, **6**:R114

- Result: 3 integrated functional networks as shown in Figure 4.

**MD5 checksum for Case Study 1**

753215e7d6fbd116b4b751f433033e87 GSE12348.dab

547f8b92a27abf7ee7e0fefbaea26a7d GSE14206.dab

de3b0b554e9fa7cda4c2d7e0cef1f44b GSE2443.dab

3f4220a6d8139d713ad99074eda1a683 GSE3868.dab

3963817e76c07e6c02659ad8197dbb53 GSE5377.dab

08904f7d70af612fe91d31ad5f733b1e GSE6956.dab

ae2b2c8168d238c361aa3cce7448e9a2 GSE7930.dab

fcdf73dcf42b3997f2b12467e3e4143e GSE8218.dab

b88dc8c8aaa786242c7d98104f866685 GSE8511.dab

0c5485652f3d39ee53c9873f6466516c GSE9347.dab

**MD5 checksum for Case Study 2**

575fa667f977f639fb50069d3e5a0792 GDS2163-GPL1261.pcl

150381b40ddd14addbb89d96a56a0efa GDS2968-GPL81.pcl

4ff3e873a2af0e6d8c9aa7e35bd7fdc4 GDS640-GPL81.pcl

0c20678d12ff95dcd0fe4d93b229af7b GSE13908.pcl

625b09bf6b5e80fbd0b88f0048ff90e7 GSE17438.pcl

9b7e4b6f0ea4d9646962f9d8b1250bd6 GSE22648.pcl

**MD5 checksum for Case Study 3**

*Bacillus subtilis:*

bbf0695a4ff2ae309922107f60d9f4e1 GSE1620.dab

b3b5d5ceceff8e6ad352c5d76a002649 GSE17435.dab

4840e395a2e62269d38c8fafde21e700 GSE17808.dab

da516613b5ab116a7ef47c34bbd277f0 GSE17809.dab

4a6ab0cd479b5f49395a6e74969b0863 GSE17884_GPL3655.dab

5b1db24f4522a810296e1eb5792683f3 GSE2667.dab

8a28d01464336aff2236103b3aff6c8c GSE4670_GPL3664.dab

3cb24da9c7661c1c09991923db5fa800 GSE4670_GPL3665.dab

c95cc5c6305baf5b91b3206fc381a590 GSE4673_GPL3645.dab

be446be42b537c7ebff9dee434b763d8 GSE4673_GPL3655.dab

c429e420d9637d7f0fc013174c7be25a GSE4673_GPL3663.dab

d8f8b8b5d579eb43231394a536fc92c4 GSE4673_GPL3668.dab

d03231a60792bb213dcb81e8d206f474 GSE4673_GPL3671.dab

4bdbcb37b67f158c9f5a9ce4d8fe7584 GSE5210_GPL3950.dab

81e0861732eb9a093b27b7be17e0fad7 GSE6865.dab

d7392d2bf1134fdf52e28259d0396cfd IntAct_taxid_224308_mapped.dab

07c39bf694266d0c81df9f4d09c827ee IntAct_taxid_224308_pmid_16796675_mapped.dab

be32e7753e47936f507ecda87a8b9108 IntAct_taxid_224308_pmid_21630458_mapped.dab

6ef2a3b6148dfb6c532d38452fa26877 MPIDB_taxid_1423_mapped.dab

287ae47bb599d2abf247d27d98b9046a MPIDB_taxid_1423_pmid_18156271_mapped.dab

5f9822a9489ecaba16130401931de1c4 PrositeDomine_224308_mapped.dab

89967dfbb1fd824c2da12eb32e46911a PrositeDomine_655816_mapped.dab

11dddea966bb18d4db674e8e2eaa05eb STRING_taxid_224308_mode_activation_mapped.dab

202798abfb2ee9b8f6c92de508042986 STRING_taxid_224308_mode_binding_mapped.dab

1afec781998537a533d8169b3e99fadd STRING_taxid_224308_mode_expression_mapped.dab

89ba34b1fd709b557e462d324f8f35a8 STRING_taxid_224308_mode_ptmod_mapped.dab

*Pseudomonas aeruginosa:*

f60e62236f2d46ecf63c70e284dc882e GDS1910-GPL84.dab

5cd2215656cdcbf92b5af6ef8c3fdc55 GDS2111-GPL84.dab

7e671ca973c7fd0fae3135186597066b GDS2317-GPL84.dab

ee967ef6aa4b84d7404ffd25ecbb1f60 GDS2377-GPL84.dab

40583ed4babeb6c6ecc447398cda56fc GDS2502-GPL84.dab

1f0a869796060fbf77aa491d079b17a0 GDS2869-GPL84.dab

cb2cfc9454c73a15287abc0c6d8efef7 GDS2870-GPL84.dab

51ef56834afc5f0dbbf51ce651ae49c2 GDS2893-GPL84.dab

09a9be513800ff85f9b014e8ccd87a09 GDS3174-GPL84.dab

f68c4052cc528a4332a20fcaf62542aa GDS3251-GPL84.dab

f8053bf1b8c2d29dc6c10e6865303cff GDS3562-GPL84.dab

1ec76be13c6c9b3888949f81d81d140a GSE10030.dab

1521f1cbdaa6b91d3531d5853f79a72a GSE10304.dab

74a9722597cb2beaf8fc7ce574924988 GSE10362.dab

3b72660832c3a17787a4c59584299f28 GSE10604.dab

87307e18edb7f9354a3934be40f89a7a GSE12207.dab

6d913d5a8ecfea419c076b85750373db GSE12738.dab

302f2e4e8b47e6506c6b2206aeb5b00d GSE13871.dab

6a6b46eb7af537c8455fc613cb0409e9 GSE14253.dab

a4b55b70447d399a021869f5dcbe3c31 GSE15697.dab

7fc5f8654ef8919f9c4cc4773c0ef477 GSE17111.dab

768dcd5baec4cac86efa7cd47e2429ca GSE17116.dab

f2768979fbc4350906e2beb14e2f2785 GSE17147.dab

a0d7851029e1c4b981f6b742b1932232 GSE17179.dab

9f13fc7caa2e2c0ad0b8f3600ae047c8 GSE17296.dab

cc688f9caac7770753eae612dacda92d GSE21508.dab

e2831c4dc3527372f41e9604d92f3162 GSE21704.dab

7fd2fa57b27c396e8a2c87fb3edb64f5 GSE21966.dab

f6922ffd175e3b1231d4c7fdbddc9cdf GSE22164.dab

0bc4e500ee64c6a662e54aaf1a18ced4 GSE22665.dab

cb6c95c86c7cf25a8d14f71f07aefa32 GSE22999.dab

19c93f3510ac9a1e230451f3fef2eb1d GSE23007.dab

c3a1fa95f42cc08d1cb098d744fc3ef3 GSE23367.dab

0e36c7a256758c549ae890be4fc1b6b9 GSE24784.dab

0c502996edf0862012578b076939d71f GSE25128.dab

baa011dc9744d34fd987623a11a456fc GSE25129.dab

bb4ab638cea2a25d806e6b1a983db569 GSE25130.dab

5fb35b12811cec35afe06df60fe8bbdf GSE25481.dab

ae7ac91e6fcaeea4c2abf82e784a44d3 GSE25595.dab

65971b583cc0748ba6f52ccddb93919c GSE25945.dab

07f6f2d7704f087a998ac5e34192cd03 GSE26142.dab

f41609261793acb5670ba1edef2e37b2 GSE27674.dab

cbc6e4483db2289d12a3bce33e102945 GSE28719.dab

bad03da3c380c3a1e64dbe14c53d57a9 GSE28953.dab

2be586279c3c30d76444fdfb48c9293c GSE29665.dab

81355350ad60fd6f4ce3d8cb5da3c5a8 GSE31227.dab

a2b661891552a88e9d4935a582c4212c GSE33188.dab

dfdb270d9dad1be976678b0e61f6cf58 GSE33275.dab

bec752b3205b09d91083d3a505fc1e77 GSE34141.dab

2dd08a51065302d55b26f983c619e233 GSE34762.dab

5b695d1e1f794c6e3af9b43794eb6612 GSE35286.dab

7a0b7a87d4b91d6d504e22318d43727e GSE36647.dab

3696cc776b06761bf98833d2ab810e52 GSE6122.dab

8a96c3b677f85b1784ec0f8cf4414ca6 GSE7968.dab

6f7745dd0b6b503e4618a51df13172b5 GSE8083.dab

3fb94d7f615bd6f12cd7fe6e202e8014 GSE8408.dab

cd28df46e2b0e36a3b8e6b4c18a56c19 GSE9255.dab

77ed9c087ebaf4601eb2efb896b11c51 GSE9592.dab

1d2279b335e8440f21190656fb5c758f GSE9621.dab

286c4206b4a7bb41d6a2e5fe0fad4c9a GSE9657.dab

2a9385c37ae53d07c9fb32a7bff6a46e GSE9926.dab

6315e60b2cd90a99894535c20febcf92 GSE9989.dab

2d08e3f2965c96a9949b413eea8d4bde GSE9991.dab

4f6664071cb9c026764b3be697c8a63f IntAct_taxid_208964.dab

1985885ce2655c686749628438c6fcc8 MPIDB_taxid_287.dab

*Escherichia coli:*

84da0d582f616861624b85f523efd21d GDS100_GPL18.dab

0009b3e9086e0ade7c296cba00c451bc GDS1804_GPL3500.dab

01a9d33558a5b69405f8ef836f61098a GDS1827_GPL199.dab

1bca33359063318972b0b45c4c28be1e GDS1848_GPL189.dab

1894a20ab291e5bc9ef3c3789f799b27 GDS1963_GPL1436.dab

635165b5642007fabcce91a82347a293 GDS2427_GPL3154.dab

516187436e0af11bcb05589e73048e50 GDS2578_GPL3503.dab

669791a0831b432d6b006b021c768270 GDS2579_GPL3503.dab

bd051f697272f99aa0705ee6b60b089d GDS2580_GPL3503.dab

64d9dddcf60b3a6d7c716a12cb8b85b8 GDS2581_GPL3503.dab

2a651dc28c9fea4a1322b86503677839 GDS2582_GPL3503.dab

25c8e0e0b0d31633b330b5d398effa83 GDS2583_GPL3503.dab

a0c402b03dae466888d7b4366251972e GDS2584_GPL3503.dab

116ca8bc896c2f5770a2ad000392b328 GDS2585_GPL3503.dab

d8db38e84d7d7efb6d86428edd94ccfb GDS2586_GPL3503.dab

135ae44cbdaf183a4222e9186257f922 GDS2587_GPL3503.dab

8c226d00d0a0ae7f70e36d1247fb45a0 GDS2588_GPL3503.dab

72f8d423b60599216858d10fd7e743c2 GDS2589_GPL3503.dab

a1747319aa35933025c99c6c6eaf6f23 GDS2590_GPL3503.dab

9c838ebd92d32461d4a5ad7e90270e62 GDS2591_GPL3503.dab

b2ffc424af03477ea91b810bee116955 GDS2592_GPL3503.dab

b7cee01dd37712015befb9bee51166ee GDS2593_GPL3503.dab

5dc1ea4cba179e16dc726068340b3a9b GDS2594_GPL3503.dab

d4ceead0d0a620c87bbc699ec3d04663 GDS2595_GPL3503.dab

ba0f9b9b72d99ec3eb84bbd77d43bf8e GDS2596_GPL3503.dab

8a9131268affee713884cf562d38ff0a GDS2597_GPL3503.dab

901dc012e7be85f97f360004b6dd61f7 GDS2598_GPL3503.dab

0a1c967005570053bff827b263754464 GDS2599_GPL3503.dab

af9478605baf02e71d16a0b4a8958223 GDS2600_GPL3503.dab

85e7d642a0bc8dbc14bf554b338772f8 GDS2753_GPL3154.dab

04591df7de0f9797d6d8e76e054c32da GDS2825_GPL189.dab

f6d973f333be8d7f02ac2cdcd8a9a714 GDS2879_GPL3154.dab

2d5bcc735594247b5ffe311f5fc93d0d GDS3123_GPL199.dab

302ee98b43068b08e77e0f872d8cb6fc GDS3421_GPL3154.dab

b9887e13150a093eb236b3723e37afa4 GDS3597_GPL3154.dab

b02c9747569edb72f9146109c403a807 GDS95_GPL16.dab

4a242a66806607253f6e8ed89f27142a GDS96_GPL17.dab

0a8c8c995476ebdf463b2dafe111b5cb GDS99_GPL17.dab

b4f6e569f9113b5c3a99c5cd694db107 GSE10116.dab

3af2237a999b10e7fb54a38be54a3f0d GSE10158.dab

a891833b72fc1b16edb471eb457ff3bc GSE10319.dab

0b1ec90f4ac54c564f35c20575f88351 GSE10345.dab

5d4b0ab39318e359535b1518e8b5e6dc GSE10440.dab

ffb9b3db7b25601248c906a0cc43ffb9 GSE11052.dab

c975ad2d13522fa30d28d95333a6e2f8 GSE11303.dab

ddef5ffb9d1cf381c99a8c7abd630858 GSE11779.dab

2e213e641b8057417297474f77f4a87c GSE11927.dab

48f2d1fd1d7e2bb47db46e6f411fc10a GSE11932.dab

3413590250b7dd68d4c37030161823ce GSE12006.dab

e8a5ec92e076b926ff935556ccf08852 GSE12190.dab

7628958c0faeb71033ea1364cacd4223 GSE12554.dab

7c8209b34e090a72f29c94351f3c6d6f GSE12701.dab

7784281d34d361639d0983823a2c8a91 GSE12750.dab

619fde48409e59e31ccb672e168e6796 GSE12797.dab

0d6b9a71c709918e54c6dab5be756290 GSE12831.dab

0875498ab0400c1bda8bc32017261714 GSE13444.dab

2292ebb972a006cd682c55293114307d GSE13562.dab

5a7fbb3cb1f6e26b2908679d996892f5 GSE13589.dab

6628eec9650427965f7daa75fe2dabf7 GSE13902.dab

f89cb36f2dd4b56732e4860599c94da8 GSE1421.dab

89c33fb960228a38cd8375235bbbeb38 GSE14472.dab

ae5669da08a78471a15fa430a689882a GSE15050.dab

753476125f1cdccf1b7935b4006fa089 GSE15059.dab

fdca5bd199efcf534aefdd1707c677f8 GSE15404.dab

6465dc22dbd791a3f7976c10030244cf GSE15405.dab

55678bbaf48bd25b54eed7806ecd52f3 GSE15406.dab

c2c2c3652f1b62c55ce34c65160bce55 GSE15500.dab

26f213c4e65cc4714a1e7c134b8870c4 GSE1730.dab

882e4ea0861d79c32eaa1a4130b58add GSE1735.dab

cdb1ef2dbe6dd5b82159bd79c4f96e33 GSE17465.dab

0d833e94e6fa6b450f35cb7693596a42 GSE17526.dab

ca6a71e02e73a8622c389e9bd7899033 GSE17584.dab

13dd718402c82b2348710f4ff7cba3e4 GSE1780.dab

b8651a9364aa1f2d5c43c470635279f9 GSE18118.dab

03b911e371a8b8e84c57094b5fb30de7 GSE18362.dab

f6f781501318efc0dbe677f646ae8805 GSE18623.dab

1ebf2317c64e18df36afd967adda9408 GSE18852.dab

efa7fbe4f9692ac3f815d6c1937cfbac GSE18972.dab

5f7f6fafb4ba949a175b3b0523005bbb GSE19655.dab

44b872b3174c43fd286bba35f7ae0f6e GSE19842.dab

0d638193bff601fab0cafa69c0c278ee GSE19879.dab

f6e00e1ebe6ef134153350a442ded146 GSE19953.dab

70ba1bc75edd85c3fb542a8968c4a7ae GSE20380.dab

4a32ce0cf56b627cbc74a7b1be13fe24 GSE21551.dab

5a900a1d8cefc6cab5dfe423a7a39b59 GSE21652.dab

699a40c0b77926674a3501610f58d661 GSE21820.dab

d0234af375b7d790271c5273b8b6cad0 GSE21893.dab

3dcd0f86233606c94be5fe467d413ecd GSE22057.dab

ba1c9dfb42b6131622dcbafd9881708f GSE23417.dab

a9dbe9b742d35a52822c4133c35d080e GSE25745.dab

daa0600be31feb8d069b8d596c5485fc GSE25982.dab

e1ffa1b8d6c7462655324145333e14d2 GSE2697.dab

173f273c81eb49306da1032a6f525e7d GSE28144.dab

fb77726c2d874b92962a6ffb117c4320 GSE28193.dab

2e93f1485f3183a3a2a1c178324f12cd GSE28412.dab

565c2abb93717c94db57d34e8f8e9482 GSE28795.dab

3e0202224f62e14d0c71ef3d052b1f94 GSE29076.dab

933e58d418ce832e62f074ecf8d5de3c GSE29439.dab

2656d18881cda5e4438b9d43a4a1324e GSE29440.dab

3b87db95233c887039a3fc52d074fdd8 GSE29486.dab

820da5fe0506299369d8d1d0d2e6dcf0 GSE29803.dab

72089f8bdb6cdf8c0de7c560026ea260 GSE29954.dab

5b2cd0fa0c48068041042e4566736514 GSE30424.dab

f7a0064eaa6f13abd75bc2e5e6a64c81 GSE30441.dab

52e99509b88aaf3cb5c8d5555cb8ba7a GSE30679.dab

4cced9e94c8819be466a59da00713e08 GSE30692.dab

d2135ab4f721088ee5083e464da95cc1 GSE30838.dab

610890fde2a928556934737bbee0ceec GSE31140.dab

2523dc208022723fbcc8f9bc6946f2c3 GSE3250_GPL2815.dab

f57c58bf9d94823b4c52ac17b1faba8c GSE3250_GPL2816.dab

d7b0d8a7c515e58c9f13c6c98806c96f GSE3250_GPL2817.dab

bc7e74c0760b56f3798ec7ee08ac17e7 GSE3250_GPL2818.dab

6f154847e6e1b63a3a29b72fddb1f28a GSE32562.dab

a0d420bfbec1da5124de19869168a9d2 GSE33.dab

dcc234c1c0dd95d7f63750d27b3dda82 GSE34028.dab

92353e9ec6cc5722b5815921b885573e GSE34046.dab

50b5900bc4d5030a39508a3ad1f5195f GSE34275.dab

d40432dc6409599d3f3a4c375fc4cdef GSE3437_GPL2928.dab

5e2c70ea75d8e7381ab9614553550cd6 GSE35100.dab

dc049d03a98c4d9d1d355a4b7eaa3185 GSE3591_GPL1246.dab

547eb58ff8457b476689ecbcc78679a1 GSE3591_GPL3051.dab

b6a74708b6ed94b93f71c2a4c3dd0b1f GSE3665.dab

4a5f02c5abb22b60b20c8be62aee6cd9 GSE36779.dab

c439d71a037a3e8347890db9a28043d5 GSE39607.dab

2270c4141aefb9e346c355badb3862fa GSE42702.dab

b41421da1c16e55bdfad66051827e034 GSE4321.dab

9aa8daed0c53bcbecd457e3a0fdfcd39 GSE4357.dab

88ebbd4bd40dc7c73457674ac0b100ea GSE4358.dab

0cd2c34d26c4a9a9589579ab502b7781 GSE4359.dab

6d4afc08fea6d291a96bb41978c9a0ba GSE4360.dab

355bb32e019b70716702118222ba2a89 GSE4361.dab

e06c2531a8f6392b995a8f657faec803 GSE4362.dab

16bcd06e2fed55fe2a90c9c419f25c32 GSE4363.dab

f41a4b0895322cd697599e2bb194f417 GSE4364.dab

cf9631c89f289034355aab8a42b0af03 GSE4365.dab

3e175679b17f562cd6468319339a88b3 GSE4366.dab

14027a0371435b6f150b8670a92d8357 GSE4367.dab

661b0afa78c2b759e4416cd4910135ac GSE4368.dab

4affa865d8e88bc246b10fbb9241505c GSE4369.dab

85c178ebe190f174fd6c6879e2d15a55 GSE4370.dab

7748ab3d9c3e0361c67b281bf4af23f6 GSE4371.dab

7684b06d075af9461ef3713e33526823 GSE4372.dab

f92152467117dba02d17318dd33afd51 GSE4373.dab

4192d0f8c6f5fb64cf22acc7989d1aab GSE4374.dab

6d63faacfc38417b3a8b841dedb55efa GSE4375.dab

79dd31a354ef436d415c9f4bbc6a2877 GSE4376.dab

996f82d15527450ee7a7e2e1cbc3f5ea GSE4377.dab

4f0a175780ce84352106d78bb5f3994c GSE4378.dab

74f7943180b22939a27d0c3398271f8e GSE4379.dab

598107564cf18625b8b19e30a165f735 GSE4380.dab

e0b4ca94279a71e551dfe688954de492 GSE4408.dab

f54bd7ec1a55c36bd79502c34c4b8532 GSE4417.dab

b16ac7d0199a31a6280bf83d7900507d GSE4511.dab

e6720ebe92e734fae4f552345e4f9536 GSE4724.dab

1ca88b42e0846565efa1b2e0c9a39485 GSE5177.dab

f663d8bf8fa2311f2178ecdac403bec9 GSE5192.dab

bb3f6fa265ba13a2deed1008537fb7ae GSE533.dab

95823abbb7c10019230779b309a5c760 GSE5552.dab

d2fc4d9fc106646f3dcd786dbe285f05 GSE6195.dab

06cec3d54ddfbfe49b2885de2468854b GSE6296.dab

5dc841dcbe113d026056ba0e30a90c1b GSE6444_GPL2928.dab

b781fb637ff8c0aaa4b6ddeff68c19b4 GSE6444_GPL3500.dab

e558d0f85bdcf20c7bece6a808bd2fd2 GSE6609.dab

c465cc0ac526cfeca99a3571d85d9bd5 GSE6712.dab

d85d64d3e900e35284b59c44361a8aed GSE6781.dab

8d38847579ad3420a26d568272242e01 GSE6992.dab

e5709f62d2a654843407aaf2dfbeafac GSE7243.dab

6c3a9ba52fc7a5e63d466e6d8e295eb8 GSE7265.dab

074c8cb07fa8d6d605480a95d6fa1e05 GSE7439.dab

554ff752529e460daf2d06e68b9b464c GSE7477.dab

f4b231e51235c3d568b318a9dad1e9fc GSE7573_GPL5113.dab

4eb95832b8cf620afb8ca386f8d3dec0 GSE7656.dab

cde09bf3460362e5353660cf3317dce8 GSE7695.dab

a8537bd002b43bb263e7fef736bd1990 GSE7931.dab

2f2dcb734f9f968394c7739c7fb25ec6 GSE7_GPL16.dab

723a5f822afbcd69b5a2bb778dfa7467 GSE7_GPL17.dab

8a98e710b948f89c3e65a1327cf09fa2 GSE8540.dab

3392f7712011ef3ab63362de3c5c46dc GSE8889.dab

3ba74138f20b227a30194fb5b45c9b1a GSE8_GPL17.dab

f6e1d64206737ba8b6d835dabf633727 GSE9178.dab

dc7ef88875f5d4674b666415c5ed1b89 GSE9388.dab

f9955c859c921600158e8f849989ca77 GSE9814.dab

cb19d20ff4226865192d160186fac026 GSE9_GPL17.dab

c15b6769c2392c89f84060085620cf9d GSE9_GPL18.dab

f893c7364f1a207b95c51a33e406d2bc IntAct_taxid_331112_mapped.dab

ef905f3bf97037bdf05faca109c45c8b IntAct_taxid_574521_pmid_12904549_mapped.dab

c46738f0a9d7dc4f136e0a1721f36187 IntAct_taxid_83333_mapped.dab

31dfd9e94f2bbfffe418c906e77f86bc IntAct_taxid_83333_pmid_15004283_mapped.dab

0a97052bbfa47c583fafd7d7247ebf14 IntAct_taxid_83333_pmid_15690043_mapped.dab

6102b9ceeccfd36808eed38671091239 IntAct_taxid_83333_pmid_15774864_mapped.dab

bdeeeda9489a4da60777d2cb22db8d79 IntAct_taxid_83333_pmid_16079137_mapped.dab

d4f1b85b17707b2b7de4fe25b131ba54 IntAct_taxid_83333_pmid_16139413_mapped.dab

58d6452bbbc8c136ca510bb3ed7750e7 IntAct_taxid_83333_pmid_16606699_mapped.dab

11216df3274bcc949e4479035b0a9d48 IntAct_taxid_83333_pmid_16858726_mapped.dab

e3e9c08fd0763b324b6242efc71ce4a2 IntAct_taxid_83333_pmid_17137328_mapped.dab

45ae807b3fb450ab015a1ea2c9369732 IntAct_taxid_83333_pmid_17938168_mapped.dab

0c2dae68e0d67f3c2a8a31eaefe5d744 IntAct_taxid_83333_pmid_19737520_mapped.dab

542fce86bb2b20873255b89f40140157 IntAct_taxid_83333_pmid_19834901_mapped.dab

b8ed6526adba07fba5b7e673ff682a47 IntAct_taxid_83333_pmid_20169075_mapped.dab

ebf99382941436c90c75007aa6eb68da MPIDB_taxid_217992_mapped.dab

30ee4bb84b7f2b774fb2f9bf2604f99d MPIDB_taxid_316385_mapped.dab

9878f195eb7e494db86b7ab3df231a97 MPIDB_taxid_331111_mapped.dab

a1a01926ee87a471b55c141119e0ee85 MPIDB_taxid_83333_mapped.dab

546055c7b416154855061ad41b751dbf MPIDB_taxid_83333_pmid_10647006_mapped.dab

e017bb165cd0c34f7a66b15e23c370ef MPIDB_taxid_83333_pmid_15522865_mapped.dab

f3f60d3ac2c7ece9cc6c57ca8b1ee7d7 MPIDB_taxid_83333_pmid_15690043_mapped.dab

54d920fc9c3ad7bdab1a38f3999c95d9 MPIDB_taxid_83333_pmid_15774864_mapped.dab

06d2cda658be2a6699977363d0b71c97 MPIDB_taxid_83333_pmid_16079137_mapped.dab

4c7c1cbad644c74a8e2b35d246ec8ff8 MPIDB_taxid_83333_pmid_16606699_mapped.dab

aac75136152c2661e8c7d932d707ec20 MPIDB_taxid_83333_pmid_16858726_mapped.dab

fc621077af5888ab30320c800f9480ac MPIDB_taxid_83333_pmid_18631241_mapped.dab

e02d67a4d9e027b04c40b6e7a7aa08ca MPIDB_taxid_83333_pmid_19834901_mapped.dab

1591de1cac6fd1d02a116bfdeeb0d1b6 MPIDB_taxid_83334_mapped.dab

eb2e4d309736d4093410590d8c055bde PrositeDomine_PrositeDomine_217992_mapped.dab

2aa4762db74bfa54063ea2cacda7d8df PrositeDomine_PrositeDomine_316385_mapped.dab

1f15fea1dacb24dc188cb80c9c016939 PrositeDomine_PrositeDomine_331111_mapped.dab

1f15fea1dacb24dc188cb80c9c016939 PrositeDomine_PrositeDomine_331112_mapped.dab

4631d02ed829f9bd3775154758a42a12 PrositeDomine_PrositeDomine_362663_mapped.dab

1d412f6f51a22e682c1c4339fb8910f1 PrositeDomine_PrositeDomine_364106_mapped.dab

eb50a6b74769e6ca68d4270dd0921f0f PrositeDomine_PrositeDomine_405955_mapped.dab

f85d0697fb537c855c8718bb199d3624 PrositeDomine_PrositeDomine_409438_mapped.dab

7a46657ae0b4e7b31223aac8768fe282 PrositeDomine_PrositeDomine_439855_mapped.dab

bfe74944ea2966e2df4293f1dc2205aa PrositeDomine_PrositeDomine_444450_mapped.dab

1015fe90dcabb5eb51a7c2ab0ca56a6f PrositeDomine_PrositeDomine_481805_mapped.dab

35290a27eeebbe64bfa1037019b869e5 PrositeDomine_PrositeDomine_574521_mapped.dab

77f00de8d11702f74fbb5dd39d1f314a PrositeDomine_PrositeDomine_585034_mapped.dab

35290a27eeebbe64bfa1037019b869e5 PrositeDomine_PrositeDomine_585035_mapped.dab

66a489a746f89b272de02f09ca1ebca6 PrositeDomine_PrositeDomine_585055_mapped.dab

d805f34a9cce55992fcca60098cc00dd PrositeDomine_PrositeDomine_585056_mapped.dab

0f051e0a6e489c514436169878cff581 PrositeDomine_PrositeDomine_585057_mapped.dab

1faec919155a816828c3851688b5c660 PrositeDomine_PrositeDomine_585397_mapped.dab

20aefeabf0a4bf6998242048b3604ded PrositeDomine_PrositeDomine_595496_mapped.dab

b04c1e23d1af2d083199bb5fda40e59e PrositeDomine_PrositeDomine_83333_mapped.dab

ad2992dac6701ef5b4d65ce74e21100d PrositeDomine_PrositeDomine_83334_mapped.dab

910d1447e85dee65cbff5fddcdba88fc STRING_taxid_155864_mode_activation_mapped.dab

9d6baee250d891e8a7bb2d8b0f5fb517 STRING_taxid_155864_mode_binding_mapped.dab

df5e3d251debf69ec4d608e4bd3e75c7 STRING_taxid_155864_mode_expression_mapped.dab

7c2906dbdba71910e67ccb3b8022b4c2 STRING_taxid_155864_mode_ptmod_mapped.dab

7f205180d73f738caaba57ecf7b3b883 STRING_taxid_199310_mode_activation_mapped.dab

c19ef297ae447606428148ed52290e01 STRING_taxid_199310_mode_binding_mapped.dab

7143c9db74c64f6fcfc44020cbf04403 STRING_taxid_199310_mode_expression_mapped.dab

621a28939b78727d6bcc64d7507e8c17 STRING_taxid_199310_mode_ptmod_mapped.dab

40883b16b9d2e2d2ea29695fdcc43aa5 STRING_taxid_316385_mode_activation_mapped.dab

0b33a569f053eb114fff2bed46ddb7b2 STRING_taxid_316385_mode_binding_mapped.dab

eedc3ea5c2ee7f899ae30c0659c0e1d0 STRING_taxid_316385_mode_expression_mapped.dab

1aa6432853d845c78452c3726c5202ff STRING_taxid_316385_mode_ptmod_mapped.dab

1ee32e7160cc851eab7bcb0e836c26a7 STRING_taxid_331111_mode_activation_mapped.dab

8e5c52f51c10e15953927c9d0aa3936d STRING_taxid_331111_mode_binding_mapped.dab

3f17aa48f0b00f4de0b0fd59277302b9 STRING_taxid_331111_mode_expression_mapped.dab

8d9f23c44e02f2110c0b0d37814b9747 STRING_taxid_331111_mode_ptmod_mapped.dab

91cbc8f6b660a826a0f1a5693df3adea STRING_taxid_331112_mode_activation_mapped.dab

64cf9f4b1728605aebd700dc4045e6c6 STRING_taxid_331112_mode_binding_mapped.dab

071e794b3e1fd6b4396902610d2431ec STRING_taxid_331112_mode_expression_mapped.dab

a71c17644c2385108f1b030d47af9be0 STRING_taxid_331112_mode_ptmod_mapped.dab

b4e8947529c3eb8986f381719bdc4251 STRING_taxid_362663_mode_activation_mapped.dab

8bc231085a5ee09748356a8c83610977 STRING_taxid_362663_mode_binding_mapped.dab

b287fd8ed86d9f7a9d2fe986aed5349d STRING_taxid_362663_mode_expression_mapped.dab

01a4b2496277748d347c7816b35b8eb6 STRING_taxid_362663_mode_ptmod_mapped.dab

270e95a294e89629c44e324321aad9c9 STRING_taxid_364106_mode_activation_mapped.dab

2d75d18fb1c03dbe1d72a6fe75a017ec STRING_taxid_364106_mode_binding_mapped.dab

ff0cdccf823bb71e037167558874e424 STRING_taxid_364106_mode_expression_mapped.dab

a26cd34cb958ffe97684fa0b80524227 STRING_taxid_364106_mode_ptmod_mapped.dab

d043942e397aece4c66875f3a78f300c STRING_taxid_386585_mode_activation_mapped.dab

54048914e28bf87cd9fb87970da55c82 STRING_taxid_386585_mode_binding_mapped.dab

5b1ebbf9903008d89ed01959af3859e5 STRING_taxid_386585_mode_expression_mapped.dab

090fcc95d5bb4bea8cb378628f8da4e2 STRING_taxid_386585_mode_ptmod_mapped.dab

370e03d0e166ee60220d81a0091f7e59 STRING_taxid_405955_mode_activation_mapped.dab

f3361264cb9f139d482366086b7b9678 STRING_taxid_405955_mode_binding_mapped.dab

6e54a8d5871a3a019cfcbb2a192d2bbd STRING_taxid_405955_mode_expression_mapped.dab

b5ccda802a36ee3dc53154b2461e7cd7 STRING_taxid_405955_mode_ptmod_mapped.dab

e4f568ee3420c29eef8149a5a8770e8f STRING_taxid_409438_mode_activation_mapped.dab

3d7a472609b720e13dc609d37f4a5b1c STRING_taxid_409438_mode_binding_mapped.dab

72998be503bc0ea9c1132f6b86054408 STRING_taxid_409438_mode_expression_mapped.dab

45f735cb26fbbd4861f7002e262a1c13 STRING_taxid_409438_mode_ptmod_mapped.dab

555cdff9dc7386723fa54daf321137dc STRING_taxid_413997_mode_activation_mapped.dab

111bd2469f21fd7d18f5b2c4b7f35bda STRING_taxid_413997_mode_binding_mapped.dab

c5d4228c1104655053825d2ad65cb7a9 STRING_taxid_413997_mode_expression_mapped.dab

08947437f7f95c7e10c8c96bac639abf STRING_taxid_413997_mode_ptmod_mapped.dab

e76864d15464cdb7ff9d82e350b79be9 STRING_taxid_439855_mode_activation_mapped.dab

0a55389e456dde149dc2d9ab51c1bf42 STRING_taxid_439855_mode_binding_mapped.dab

db49944bda1f35f9b8ed338638644ffb STRING_taxid_439855_mode_expression_mapped.dab

21133bef732267d628ef62fa0b72000d STRING_taxid_439855_mode_ptmod_mapped.dab

315278917d1221d577c29e975a4e4650 STRING_taxid_444450_mode_activation_mapped.dab

b426f197bc68c689bd0f2d63ad8ed440 STRING_taxid_444450_mode_binding_mapped.dab

665dfb03862e3a47c37b1805412758a4 STRING_taxid_444450_mode_expression_mapped.dab

54b168b80238135a0770e92ba45ded0b STRING_taxid_444450_mode_ptmod_mapped.dab

66287545eecabe20a6aeb259f64969d4 STRING_taxid_481805_mode_activation_mapped.dab

a06935c6d7e0a9b3d61048899d2b746c STRING_taxid_481805_mode_binding_mapped.dab

44a081e65ebed65a00b3d6fc9e419c73 STRING_taxid_481805_mode_expression_mapped.dab

5cb2c86fb3f62433d1021faf985782fe STRING_taxid_481805_mode_ptmod_mapped.dab

adeafb399fbae95cd9347a60f94df4d8 STRING_taxid_511145_mode_activation_mapped.dab

8d9dbcb2041e89bd471195f76e33bd5f STRING_taxid_511145_mode_binding_mapped.dab

7b71f0911bc638b1b14b21998660c25b STRING_taxid_511145_mode_expression_mapped.dab

3f8437d3c6b6c764572dffc49476a797 STRING_taxid_511145_mode_ptmod_mapped.dab

164751b88e3ae2bcf63aa0db56099953 STRING_taxid_544404_mode_activation_mapped.dab

9cd14023b90f90461a0f6886462d608c STRING_taxid_544404_mode_binding_mapped.dab

b00544f66054e07349e02aa6dbd637a5 STRING_taxid_544404_mode_expression_mapped.dab

edd329aec7f971d6149eb008dc5a70cc STRING_taxid_544404_mode_ptmod_mapped.dab

70be67c7b9be6e72933797422d668543 STRING_taxid_573235_mode_activation_mapped.dab

563ce6eb948e25a92bb999809cc3a51c STRING_taxid_573235_mode_binding_mapped.dab

d4d06f2eafc63f9aa1b95093ec991053 STRING_taxid_573235_mode_expression_mapped.dab

71cbad4bc4c6a73dad18340cd4bd923f STRING_taxid_573235_mode_ptmod_mapped.dab

4490b7545d3d1529ea2e6ea4f7bd74e9 STRING_taxid_574521_mode_activation_mapped.dab

03689ab3a178fc5a2e9b11b4e107a8f5 STRING_taxid_574521_mode_binding_mapped.dab

0db1e4fbe8c5365d24d045df28b8d144 STRING_taxid_574521_mode_expression_mapped.dab

61fa12ecfcdd658103b3dec0454127df STRING_taxid_574521_mode_ptmod_mapped.dab

63f2cb2af61c0d809a76ba1d1efc27fa STRING_taxid_585055_mode_activation_mapped.dab

a2294f5cb1ba9d26f9f8cf6d65dcc37a STRING_taxid_585055_mode_binding_mapped.dab

9369acbb41b23c82fb8387692deedb20 STRING_taxid_585055_mode_expression_mapped.dab

911e52ac782c02f8160ea2d6b5fac8a8 STRING_taxid_585055_mode_ptmod_mapped.dab

9cd9af394f5a7c7248579d68660284fc STRING_taxid_585395_mode_activation_mapped.dab

be43a014202f1cb6fdb52d097903a65f STRING_taxid_585395_mode_binding_mapped.dab

d8d5a7aea0329ff83bd96296e24d0d59 STRING_taxid_585395_mode_expression_mapped.dab

f11f5f08131959940af3dc68fb3cfabb STRING_taxid_585395_mode_ptmod_mapped.dab

e5f8250a571f088e1a60ebf86297ac47 STRING_taxid_585396_mode_activation_mapped.dab

c2b8502de65371fd3e06ef91f9546f2f STRING_taxid_585396_mode_binding_mapped.dab

7abd6572447c6a2d88ba4ec50656b538 STRING_taxid_585396_mode_expression_mapped.dab

aaa60449deeda3205c31199c8d2c0adc STRING_taxid_585396_mode_ptmod_mapped.dab

1bcf7919a643dbf3a3007a8416fce6af STRING_taxid_595496_mode_activation_mapped.dab

1a72f5904acb2614ab6926e30f817b79 STRING_taxid_595496_mode_binding_mapped.dab

af78d63636a9b57c7e7ca55bce70a340 STRING_taxid_595496_mode_expression_mapped.dab

ddbf1f0ffe2265eb2f27b80829231c8a STRING_taxid_595496_mode_ptmod_mapped.dab
