# Supplementary material for: A reproducible approach to high-throughput biological data acquisition and integration
Source: PeerJ. 2015 Mar 31;3:e791. doi: 10.7717/peerj.791 (PMC4493686; doi:10.7717/peerj.791)
Supplement: Text S2 [file peerj-03-791-s008.docx]

**Supplementary Text 2: Metadata file example**

One of ARepA's main outputs are metadata files in the python pickle (pkl) format; the pickle module implements an algorithm for serializing and de-serializing a python object structure using a printable ASCII representation without restrictions imposed by external standards.

To read the metadata (pkl) format, ARepA provides a script to de-serialize the file which can be found in the source directly: arepa/src/unpickle.py

$ python arepa/src/unpickle.py < metadata.pkl

Example from the prostate cancer case study (Supplementary Table S1): GSE12348

$ python arepa/src/unpickle.py < GSE12348.pkl

Output:

|  | ['GSM310067', 'GSM310069', 'GSM310080', 'GSM310081', 'GSM310082', 'GSM310083', 'GSM310084', 'GSM310120', 'GSM310121'] |
| --- | --- |
| label_ch1 | ['biotin', 'biotin', 'biotin', 'biotin', 'biotin', 'biotin', 'biotin', 'biotin', 'biotin'] |
| Series_platform_taxid | 9606 |
| label_protocol_ch1 | ["Affymetrix U133A manufacturer's recommended protocol", "Affymetrix hgU133a manufacturer's recommended protocol", "Affymetrix hgU133a manufacturer's recommended protocol", "Affymetrix hgU133a manufacturer's recommended protocol", "Affymetrix hgU133a manufacturer's recommended protocol", "Affymetrix hgU133a manufacturer's recommended protocol", "Affymetrix hgU133a manufacturer's recommended protocol", "Affymetrix hgU133a manufacturer's recommended protocol", "Affymetrix hgU133a manufacturer's recommended protocol"] |
| data_processing | ['MAS5.0', 'MAS5.0', 'MAS5.0', 'MAS5.0', 'MAS5.0', 'MAS5.0', 'MAS5.0', 'MAS5.0', 'MAS5.0'] |
| channels | 1 |
| contact_address | ['1650 Orleans Street, CRB1 Rm 1M53', '1650 Orleans Street, CRB1 Rm 1M53', '1650 Orleans Street, CRB1 Rm 1M53', '1650 Orleans Street, CRB1 Rm 1M53', '1650 Orleans Street, CRB1 Rm 1M53', '1650 Orleans Street, CRB1 Rm 1M53', '1650 Orleans Street, CRB1 Rm 1M53', '1650 Orleans Street, CRB1 Rm 1M53', '1650 Orleans Street, CRB1 Rm 1M53'] |
| Series_contact_name | Srinivasan,,Yegnasubramanian |
| Series_status | Public on Nov 01 2008 |
| type | ['RNA', 'RNA', 'RNA', 'RNA', 'RNA', 'RNA', 'RNA', 'RNA', 'RNA'] |
| gloss | The primary goal of this study was to assess differences in gene expression between prostate cancer cell lines and normal prostate epithelial and stromal cells in primary culture. |
| contact_name | ['Srinivasan,,Yegnasubramanian', 'Srinivasan,,Yegnasubramanian', 'Srinivasan,,Yegnasubramanian', 'Srinivasan,,Yegnasubramanian', 'Srinivasan,,Yegnasubramanian', 'Srinivasan,,Yegnasubramanian', 'Srinivasan,,Yegnasubramanian', 'Srinivasan,,Yegnasubramanian', 'Srinivasan,,Yegnasubramanian'] |
| source_name_ch1 | ['1s normal prostate stromal cells, basal conditions', '4ST prostate normal stromal cells', 'C42B prostate cancer cell line', 'CWR22Rv1 prostate cancer cell line', 'DU-145 prostate cancer cell line', 'LAPC4 prostate cancer cells', 'LNCaP prostate cancer cells', 'PC-3 prostate cancer cell line', 'PrEC normal prostate epithelial cells'] |
| supplementary_file.1 | ['ftp://ftp.ncbi.nlm.nih.gov/pub/geo/DATA/supplementary/samples/GSM310nnn/GSM310067/GSM310067.CHP.gz', 'ftp://ftp.ncbi.nlm.nih.gov/pub/geo/DATA/supplementary/samples/GSM310nnn/GSM310069/GSM310069.CHP.gz', 'ftp://ftp.ncbi.nlm.nih.gov/pub/geo/DATA/supplementary/samples/GSM310nnn/GSM310080/GSM310080.CHP.gz', 'ftp://ftp.ncbi.nlm.nih.gov/pub/geo/DATA/supplementary/samples/GSM310nnn/GSM310081/GSM310081.CHP.gz', 'ftp://ftp.ncbi.nlm.nih.gov/pub/geo/DATA/supplementary/samples/GSM310nnn/GSM310082/GSM310082.CHP.gz', 'ftp://ftp.ncbi.nlm.nih.gov/pub/geo/DATA/supplementary/samples/GSM310nnn/GSM310083/GSM310083.CHP.gz', 'ftp://ftp.ncbi.nlm.nih.gov/pub/geo/DATA/supplementary/samples/GSM310nnn/GSM310084/GSM310084.CHP.gz', 'ftp://ftp.ncbi.nlm.nih.gov/pub/geo/DATA/supplementary/samples/GSM310nnn/GSM310120/GSM310120.CHP.gz', 'ftp://ftp.ncbi.nlm.nih.gov/pub/geo/DATA/supplementary/samples/GSM310nnn/GSM310121/GSM310121.CHP.gz'] |
| Series_contact_state | MD |
| Series_contact_address | 1650 Orleans Street, CRB1 Rm 1M53 |
| title | ['1S_prostate_normal_stromacells-untreated', '4ST_prostate_normal_stromalcells-untreated', 'C42B_prostate_cancer_cell_line-untreated', 'CWR22Rv1_prostate_cancer_cell_line-untreated', 'DU145_prostate_cancer_cell_line-untreated', 'LAPC4_prostate_cancer_cell_line-untreated', 'LNCaP_prostate_cancer_cell_line-untreated', 'PC3_prostate_cancer_cell_line-untreated', 'PREC_prostate_normal_epithelial_cells-untreated'] |
| scan_protocol | ["Affymetrix U133A manufacturer's recommended protocol", "Affymetrix hgU133a manufacturer's recommended protocol", "Affymetrix hgU133a manufacturer's recommended protocol", "Affymetrix hgU133a manufacturer's recommended protocol", "Affymetrix hgU133a manufacturer's recommended protocol", "Affymetrix hgU133a manufacturer's recommended protocol", "Affymetrix hgU133a manufacturer's recommended protocol", "Affymetrix hgU133a manufacturer's recommended protocol", "Affymetrix hgU133a manufacturer's recommended protocol"] |
| contact_institute | ['Johns Hopkins University', 'Johns Hopkins University', 'Johns Hopkins University', 'Johns Hopkins University', 'Johns Hopkins University', 'Johns Hopkins University', 'Johns Hopkins University', 'Johns Hopkins University', 'Johns Hopkins University'] |
| Series_contact_city | Baltimore |
| platform | GPL96 |
| treatment_protocol_ch1 | ['No treatment', 'Untreated', 'Untreated', 'Untreated', 'Untreated', 'Untreated', 'Untreated', 'Untreated', 'Untreated'] |
| pmid | 18974140 |
| conditions | 9 |
| Series_overall_design | 9 samples were analyzed with 1 replicate each. Samples included 6 prostate cancer cell lines, 1 normal prostate epithelial cell system in primary culture, and 2 normal prostate stromal cell systems in primary culture. |
| submission_date | ['Aug 05 2008', 'Aug 05 2008', 'Aug 05 2008', 'Aug 05 2008', 'Aug 05 2008', 'Aug 05 2008', 'Aug 05 2008', 'Aug 05 2008', 'Aug 05 2008'] |
| extract_protocol_ch1 | ['Qiagen RNeasy kit', 'Qiagen RNeasy kit', 'Qiagen RNeasy kit', 'Qiagen RNeasy kit', 'Qiagen RNeasy kit', 'Qiagen RNeasy kit', 'Qiagen RNeasy kit', 'Qiagen RNeasy kit', 'Qiagen RNeasy kit'] |
| contact_state | ['MD', 'MD', 'MD', 'MD', 'MD', 'MD', 'MD', 'MD', 'MD'] |
| description | ['None', 'None', 'None', 'None', 'None', 'None', 'None', 'None', 'None'] |
| taxid_ch1 | ['9606', '9606', '9606', '9606', '9606', '9606', '9606', '9606', '9606'] |
| Series_contributor | Srinivasan,,Yegnasubramanian  William,G,Nelson |
| biomaterial_provider_ch1 | ['John T. Isaacs laboratory', 'John T. Isaacs Laboratory', 'John T. Isaacs Laboratory', 'ATCC', 'ATCC', 'John T. Isaacs laboratory', 'ATCC', 'ATCC', 'Cambrex'] |
| Series_submission_date | Aug 05 2008 |
| channel_count | ['1', '1', '1', '1', '1', '1', '1', '1', '1'] |
| characteristics_ch1 | ['These are a batch of normal prostate stromal cells propagated in primary culture. These cells are not immortalized.', 'Normal stromal cells from a the prostate gland grown in primary culture. Not transformed or immortalized.', 'This is a prostate cancer cell line that is an androgen independent variant of the LNCaP prostate cancer cell line. It is directly derived from the LNCaP cell line.', 'CWR22Rv1 prostate cancer cell line', 'Androgen receptor negative prostate cancer cell line', 'Androgen sensitive', 'Androgen sensitive prostate cancer cell line', 'PC3 androgen receptor negative prostate cancer cell line', 'Normal prostate epithelial cells grown in primary culture'] |
| geo_accession | ['GSM310067', 'GSM310069', 'GSM310080', 'GSM310081', 'GSM310082', 'GSM310083', 'GSM310084', 'GSM310120', 'GSM310121'] |
| Series_contact_zip/postal_code | 21231 |
| last_update_date | ['Sep 24 2008', 'Sep 24 2008', 'Sep 24 2008', 'Sep 24 2008', 'Sep 24 2008', 'Sep 24 2008', 'Sep 24 2008', 'Sep 24 2008', 'Sep 24 2008'] |
| growth_protocol_ch1 | ['Cells were propagated in Iscove\xe2\x80\x99s media (Life Technologies, Inc.) containing 10% fetal bovine serum and 1 nM R1881', "Grown/propagated in Iscove's media with 10% FBS and 1nM R1881", 'Grown/propagated in RPMI 1640 with 10% FBS.', 'Grown/propagated in RPMI 1640 with 10% FBS.', 'Grown/propagated in RPMI 1640 with 10% FBS.', 'Grown/propagated in RPMI 1640 with 10% FBS and 1 nM R1881', 'Grown/propagated in RPMI 1640 with 10% FBS', 'Grown/propagated in RPMI 1640 with 10% FBS', 'Grown/propagated in RPMI 1640 with 10% FBS'] |
| Series_supplementary_file | ftp://ftp.ncbi.nlm.nih.gov/pub/geo/DATA/supplementary/series/GSE12348/GSE12348_RAW.tar |
| Series_relation | BioProject: http://www.ncbi.nlm.nih.gov/bioproject/113173 |
| molecule_ch1 | ['total RNA', 'total RNA', 'total RNA', 'total RNA', 'total RNA', 'total RNA', 'total RNA', 'total RNA', 'total RNA'] |
| Series_contact_country | USA |
| data_row_count | ['22283', '22283', '22283', '22283', '22283', '22283', '22283', '22283', '22283'] |
| curated | ['', 'title', 'geo_accession', 'status', 'submission_date', 'last_update_date', 'type', 'channel_count', 'source_name_ch1', 'organism_ch1', 'characteristics_ch1', 'biomaterial_provider_ch1', 'treatment_protocol_ch1', 'growth_protocol_ch1', 'molecule_ch1', 'extract_protocol_ch1', 'label_ch1', 'label_protocol_ch1', 'taxid_ch1', 'hyb_protocol', 'scan_protocol', 'description', 'data_processing', 'platform_id', 'contact_name', 'contact_institute', 'contact_address', 'contact_city', 'contact_state', 'contact_zip/postal_code', 'contact_country', 'supplementary_file', 'supplementary_file.1', 'data_row_count'] |
| contact_city | ['Baltimore', 'Baltimore', 'Baltimore', 'Baltimore', 'Baltimore', 'Baltimore', 'Baltimore', 'Baltimore', 'Baltimore'] |
| Series_geo_accession | GSE12348 |
| Series_sample_id | GSM310067 GSM310069 GSM310080 GSM310081 GSM310082 GSM310083 GSM310084 GSM310120 GSM310121 |
| taxid | 9606 |
| platform_id | ['GPL96', 'GPL96', 'GPL96', 'GPL96', 'GPL96', 'GPL96', 'GPL96', 'GPL96', 'GPL96'] |
| status | ['Public on Nov 01 2008', 'Public on Nov 01 2008', 'Public on Nov 01 2008', 'Public on Nov 01 2008', 'Public on Nov 01 2008', 'Public on Nov 01 2008', 'Public on Nov 01 2008', 'Public on Nov 01 2008', 'Public on Nov 01 2008'] |
| contact_country | ['USA', 'USA', 'USA', 'USA', 'USA', 'USA', 'USA', 'USA', 'USA'] |
| mapped | TRUE |
| supplementary_file | ['ftp://ftp.ncbi.nlm.nih.gov/pub/geo/DATA/supplementary/samples/GSM310nnn/GSM310067/GSM310067.CEL.gz', 'ftp://ftp.ncbi.nlm.nih.gov/pub/geo/DATA/supplementary/samples/GSM310nnn/GSM310069/GSM310069.CEL.gz', 'ftp://ftp.ncbi.nlm.nih.gov/pub/geo/DATA/supplementary/samples/GSM310nnn/GSM310080/GSM310080.CEL.gz', 'ftp://ftp.ncbi.nlm.nih.gov/pub/geo/DATA/supplementary/samples/GSM310nnn/GSM310081/GSM310081.CEL.gz', 'ftp://ftp.ncbi.nlm.nih.gov/pub/geo/DATA/supplementary/samples/GSM310nnn/GSM310082/GSM310082.CEL.gz', 'ftp://ftp.ncbi.nlm.nih.gov/pub/geo/DATA/supplementary/samples/GSM310nnn/GSM310083/GSM310083.CEL.gz', 'ftp://ftp.ncbi.nlm.nih.gov/pub/geo/DATA/supplementary/samples/GSM310nnn/GSM310084/GSM310084.CEL.gz', 'ftp://ftp.ncbi.nlm.nih.gov/pub/geo/DATA/supplementary/samples/GSM310nnn/GSM310120/GSM310120.CEL.gz', 'ftp://ftp.ncbi.nlm.nih.gov/pub/geo/DATA/supplementary/samples/GSM310nnn/GSM310121/GSM310121.CEL.gz'] |
| Series_last_update_date | Nov 14 2012 |
| organism_ch1 | ['Homo sapiens', 'Homo sapiens', 'Homo sapiens', 'Homo sapiens', 'Homo sapiens', 'Homo sapiens', 'Homo sapiens', 'Homo sapiens', 'Homo sapiens'] |
| hyb_protocol | ["Affymetrix U133A manufacturer's recommended protocol", "Affymetrix hgU133a manufacturer's recommended protocol", "Affymetrix hgU133a manufacturer's recommended protocol", "Affymetrix hgU133a manufacturer's recommended protocol", "Affymetrix hgU133a manufacturer's recommended protocol", "Affymetrix hgU133a manufacturer's recommended protocol", "Affymetrix hgU133a manufacturer's recommended protocol", "Affymetrix hgU133a manufacturer's recommended protocol", "Affymetrix hgU133a manufacturer's recommended protocol"] |
| Series_contact_institute | Johns Hopkins University |
| contact_zip/postal_code | ['21231', '21231', '21231', '21231', '21231', '21231', '21231', '21231', '21231'] |
